# Validation of HemoCue method and development of a revised hemoglobin cut-off to detect anemia in children aged 6–24-months

**DOI:** 10.1371/journal.pone.0346506

**Published:** 2026-10-16

**Authors:** Mustafa Mahfuz, Ar-Rafi Khan, S. M. Tafsir Hasan, Md. Shabab Hossain, A. H. M. Rezwan, Mehjabin Tishan Mahfuz, Md. Ashraful Alam, Tahmeed Ahmed

**Affiliations:** 1 Nutrition Research Division, International Centre for Diarrhoeal Disease Research, Bangladesh (icddr, b), Dhaka, Bangladesh; 2 Office of Executive Director, International Centre for Diarrhoeal Disease Research, Bangladesh (icddr, b), Dhaka, Bangladesh; Kintampo Health Research Centre, GHANA

## Abstract

**Background:**

Accurate hemoglobin (Hb) measurement is essential for determining anemia prevalence at the population level. The HemoCue method, commonly used in resource poor settings, has been shown to overestimate Hb levels compared to the direct cyanmethemoglobin (DCM) method, leading to an underestimation of anemia. We aimed to validate the HemoCue method against the DCM method and establish an optimal cut-off value for diagnosing anemia in children under two.

**Methods:**

Data were collected from children under two years of age as part of the Etiology, Risk Factors, and Interactions of Enteric Infections and Malnutrition and the Consequences for Child Health and Development study in Bangladesh, conducted in Mirpur, Dhaka, from February 2010 to February 2017. Using HemoCue 201 instrument and DCM method, Hb levels were measured in 846 venous blood samples collected from 589 children. Quadratic regression was explored to derive a correction factor; however, substantial dispersion in HemoCue measurements prevented establishment of a reliable correction model. Youden’s Index was used to identify the optimal cut‑off. While it provided a strong threshold, we prioritized achieving at least 70% sensitivity and specificity to confirm balanced diagnostic performance.

**Results:**

The median (IQR) Hb concentrations estimated by HemoCue and DCM were 11.6 (10.5, 12.5) g/dL and 10.8 (10.1, 11.5) g/dL, respectively. Using a cut-off of <10.5 g/dL for anemia, the prevalence was significantly higher with the DCM method (35.2% vs. 23.2%, p < 0.001). Considering DCM as the gold standard, the sensitivity and specificity of the HemoCue method were 93% and 53%, respectively. In the test/train analysis, the newly identified cut-off of 11.00 g/dL, indicated an anemia prevalence of 34.2% in the testing dataset, with a sensitivity of 84% and specificity of 69%.

**Conclusion:**

This study highlights substantial variability between HemoCue and DCM hemoglobin measurements, which prevented development of a reliable correction factor. The proposed HemoCue cut-off of 11.0 g/dL may improve anemia detection in children under two years of age when compared with the currently recommended threshold.

## Introduction

Anemia is a major public health issue, contributing to chronic infections and nonspecific diseases [1,2]. Globally, around 1.8 billion people are affected by anemia, with a prevalence of 39.8% in children under five years old [3–6]. The consequences of anemia include impaired mental and physical growth, altered social behavior, and reduced cognitive performance [3,7,8]. These impacts can extend to poor educational outcomes and, over time, harm a country’s economic growth, with an estimated cost ranging from $7,000 to $29,000 USD per case and up to 4% of GDP loss due to iron deficiency, the leading cause of anemia [9–12].

The World Health Organization (WHO) defines anemia in children under two as a hemoglobin (Hb) concentration below 10.5 g/dL [13]. Hb concentration is the most common indicator for anemia, and the cyanmethemoglobin method is the gold standard recommended by the International Committee for Standardization in Hematology [14–16]. Although automated hematology analyzers are accurate, they are expensive and impractical for large-scale or field-based studies [17]. The HemoCue photometric method offers a more portable, cost-effective alternative, providing digital Hb readings within 10 seconds using a small blood sample without requiring electricity or refrigeration [16,18,19]. This device is portable, user-friendly, and time-efficient, making it ideal for fieldwork [16,18,20]. However, previous studies comparing HemoCue with reference laboratory methods have reported variable results, with differences depending on factors such as specimen type, analytical procedures, study setting, and HemoCue model [14,21,22]. Although many studies have found that HemoCue measurements tend to yield higher hemoglobin values and lower estimates of anemia prevalence, substantial heterogeneity has been documented across investigations [14]. Such variability may influence population-level estimates of anemia and warrants evaluation within specific settings and populations. This discrepancy in Hb estimation may lead to an underestimation of anemia prevalence at the population level.

This study, conducted in Dhaka's urban slums, compares the sensitivity and specificity of the HemoCue method against the cyanmethemoglobin method using venous blood samples in children under two. The paper aims to validate the HemoCue method in comparison to the cyanmethemoglobin standard and proposes a revised hemoglobin cut-off value for detecting anemia in this population.

## Methods

This study utilized hemoglobin (Hb) data from children under two years of age, collected as part of the Etiology, Risk Factors, and Interactions of Enteric Infections and Malnutrition and the Consequences for Child Health and Development (MAL-ED) study in Bangladesh (PR-2008–020) (ClinicalTrials.gov Identifier: NCT02441426), conducted in Mirpur, Dhaka, from February 2010 to February 2017. Data for the present study was collected between 04 October 2010 and 05 June 2013. The primary objective of the study was to explore the relationships between various risk factors for malnutrition, gut physiology, microbial exposure, infections, diet, nutrition, and their impacts on physical and cognitive development as well as immune responses [23]. Comprehensive descriptions of the MAL-ED study and its Bangladesh site have been published elsewhere [23].

The Bangladesh MAL-ED study comprised two main components: (i) a birth cohort study and (ii) a case-control study. In the birth cohort component, children were enrolled within 17 days of birth, following clearly defined inclusion and exclusion criteria, and were followed until five years of age. Blood samples were collected at 7, 15, 24, 36, and 60 months. In contrast, the case-control component was a prospective community-based intervention study that enrolled two groups of children aged 6–24 months and followed them for five months. The case group consisted of severely to moderately underweight children (weight-for-age z-score < −2, based on the WHO 2006 growth reference), while the control group included age- and community-matched well-nourished children (WAZ > −1). In the case-control study, venous blood samples were collected at enrollment and five months after enrollment. Hemoglobin levels were measured in these samples using the HemoCue 201 instrument (HemoCue AB, Ängelholm, Sweden) and the direct cyanmethemoglobin (DCM) method [23].

For the present analysis, we included only children with paired hemoglobin measurements available from both the HemoCue and DCM methods. Participants without paired measurements were excluded from the analysis. Missing paired measurements were primarily attributable to loss to follow-up, refusal of blood collection, and clotting of blood specimens that precluded DCM analysis despite successful HemoCue measurement. Consequently, the analytical sample represents a subset of participants enrolled in the original MAL-ED studies.

### Blood collection, processing, and storage

Approximately 5 mL of venous blood was collected from each of the 589 participating child using a butterfly needle system, following a standardized operating procedure approved by the ethical review board. Hemoglobin concentration was measured immediately after blood collection using the HemoCue 201 system. Following collection, blood samples were placed in coolers with cold packs, centrifuged within two hours, transported to the icddr,b laboratory under controlled conditions, and stored at –80°C until further laboratory analyses, including DCM measurement [24,25].

### Hemoglobin measurement

Hemoglobin concentration was measured immediately after blood collection using a HemoCue 201 instrument (HemoCue AB, Ängelholm, Sweden) [26]. This determines hemoglobin concentration photometrically after conversion of hemoglobin to azidemethemoglobin within a reagent-containing microcuvette. In addition, hemoglobin concentration was measured using the direct cyanmethemoglobin (DCM) method, which is considered the reference standard for total hemoglobin measurement. In this method, blood samples were mixed with Drabkin's reagent containing potassium ferricyanide and potassium cyanide, converting hemoglobin to a stable cyanmethemoglobin derivative that was subsequently quantified spectrophotometrically at 540 nm [27]. To ensure analytical quality, control samples with assigned hemoglobin concentrations were analyzed in duplicate during each analytical run. The assay demonstrated good analytical performance, with within-day coefficients of variation below 2%, between-day coefficients of variation below 3%, and recovery values ranging from 97% to 102%. Although the exact interval between HemoCue and DCM measurements was not recorded, all specimens were processed, transported, and stored according to standardized procedures to preserve sample integrity prior to laboratory analysis.

### Statistical analysis plan

Data were summarized either as means and standard deviations (SD) or median (inter quartile range (IQR)), as appropriate. Anemia diagnosis was made using a cut-off point of <10.50 g/dl for Hb recommended by the WHO [13]. Hemoglobin measurements from venous blood using the DCM method were considered the gold standard for diagnosing anemia. Both the HemoCue and DCM methods were used to diagnose anemia based on venous samples. Inter-rater reliability for anemia diagnosis between the two methods was assessed using Cohen's kappa statistic. McNemar’s test was employed for paired anemia data, and Pearson's correlation coefficient was calculated using the pairwise method [28].

Agreement between the DCM and HemoCue methods was evaluated using the Bland–Altman plot [29]. An initial ordinary least squares (OLS) linear regression model was fitted with DCM hemoglobin concentration as the dependent variable and HemoCue hemoglobin concentration as the independent variable to assess whether a correction factor could be derived. Model assumptions were evaluated using scatter plots and visual inspection of residual patterns, with particular emphasis on the assumption of linearity. Because substantial dispersion in HemoCue measurements was observed across the range of DCM values, the linearity assumption was not satisfied. A quadratic regression model was subsequently explored to improve model fit; however, a reliable correction factor could not be established. Therefore, no correction factor is recommended.

Because the objective of the regression analyses was to evaluate the functional relationship between HemoCue and DCM measurements rather than estimate the effects of participant characteristics, no covariate adjustments were included in these models. Furthermore, because both measurements were obtained from the same individuals at the same time point, participant-level characteristics that could potentially confound the comparison were inherently controlled for in the study design.

Sensitivity, specificity, positive predictive value (PPV), and negative predictive value (NPV) were estimated for diagnosing anemia using the HemoCue method. A receiver operating characteristic (ROC) curve analysis was performed to assess Hb levels measured by the HemoCue method in relation to anemia with the area under curve calculated to assess classification performance [30]. Receiver operating characteristic (ROC) analysis was performed using continuous HemoCue hemoglobin values as the test variable and anemia status defined by the DCM reference method (Hb < 10.5 g/dL) as the classification outcome. Youden’s Index was used to identify the optimal cut-off point for Hb measured by the HemoCue method [31]. To ensure a clinically balanced diagnostic cut-off, we did not rely solely on Youden’s Index. Instead, we used a minimum sensitivity and specificity threshold of 70% as an initial screening criterion to identify candidate cut-off values with acceptable diagnostic performance. Final cut-off selection was based on a combination of sensitivity, specificity, balanced accuracy, agreement statistics, and clinical interpretability [32]. Nearby alternative thresholds were also assessed to determine whether any cut-off points provided meaningful improvements in terms of statistical robustness and biological relevance.

To ensure adequate precision, particularly sensitivity and specificity, we performed the required sample size calculation as part of power analysis. With a 5% significance level and precision of 5% for both sensitivity and specificity, the following formulas were used to determine the required sample size:

Sensitivity:


$$\begin{array}{c} n_{\text{disease}}=\frac{Z^{\text{2}}\cdot Se\cdot (\text{1}-Se)}{d^{\text{2}}} \end{array}$$

Specificity:


$$\begin{array}{c} n_{\text{non}-\text{disease}}=\frac{Z^{\text{2}}\cdot Sp\cdot (\text{1}-Sp)}{d^{\text{2}}} \end{array}$$

Where:

Z = Z-score for the 95% confidence level

*Se* = Expected sensitivity

*Sp* = Expected specificity

*d* = Desired precision 5%

The sample size was calculated to estimate the respective proportions. The total sample size was adjusted to account for disease prevalence. This approach ensures that the study is sufficiently powered and provide reliable estimates of both sensitivity and specificity.

To internally validate the performance of the proposed HemoCue hemoglobin cut-off, the dataset was randomly divided into training (70%) and testing (30%) sets. The training dataset was used to identify candidate cut-off values, whereas the testing dataset was used to evaluate their diagnostic performance in an independent subset of the study data.

Exploratory subgroup analyses were conducted by stratifying participants based on age, nutritional status and inflammation status. Participants aged between 6–12 were considered as infant while toddler were aged between 12–24 months. Stunted children were defined as length-for-age ≤ –2 SD. Underweight children were defined as weight-for-age ≤ –2 SD. Acute systemic inflammation status were identified based on C-reactive protein (CRP) cut-off of ≥ 5 mg/L and Alpha-1-Acid Glycoprotein (AGP) cut-off of ≥ 100 mg/dL [33,34]. All statistical analyses were conducted using R software (version 4.5.2).

### Ethics

The Ethical Review Committee (ERC) of the International Centre for Diarrhoeal Diseases and Research, Bangladesh (icddr,b), has approved the study, with the protocol number PR-2008–020. Before participation in the study, one of the parents or legal guardians of the participants provided written informed consent, affirming their willingness to participate after being fully informed about the study's nature, purpose, risks, and benefits.

## Results

A total of 265 healthy newborns were enrolled in the birth cohort study within 17 days of birth, following strict inclusion criteria. Plasma samples were collected at 7 and 15 months. Hemoglobin was measured using both the DCM and HemoCue methods with 173 participants assessed at 7 months and 165 participants assessed at 15 months. In the case-control component of the MAL-ED study, 500 cases and 480 controls were enrolled. Hemoglobin data from both DCM and HemoCue methods were available for 87 cases and 178 controls at baseline, and for 99 cases and 144 controls at the endline (5 months post-enrollment). Overall, 589 children contributed 846 paired hemoglobin assessments from venous blood using both the HemoCue and DCM methods (Fig 1). Baseline data for birth cohort were derived from the six‑month surveillance visit, which represented the closest available round to the scheduled SES. No differences were observed in birth cohort (S1 Table). In contrast, in the case control cohorts, participants with available measurements weight, height and handwashing with soap before preparing food were significantly lower to those without available measurements (S2 and S3 Tables).

**Fig 1:**
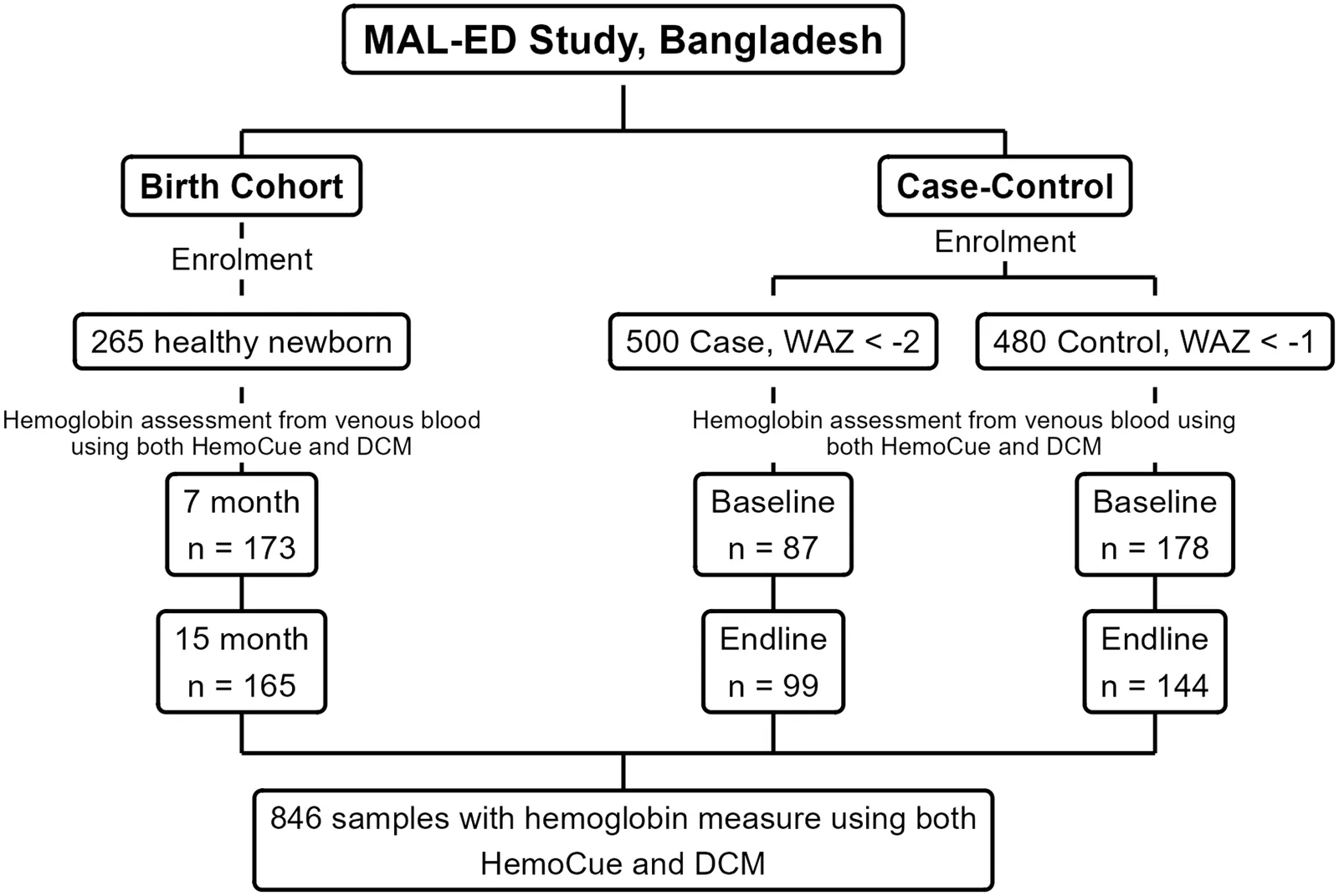
Sample selection flow chart from different cohorts used in the analysis.

In the MaL-ED birth cohort study, 265 healthy newborns were enrolled within 17 days of birth. Plasma samples were collected at 7 and 15 months. Hemoglobin levels were measured using both the direct cyanmethemoglobin (DCM) and HemoCue methods for 173 and 165 participants, respectively. In the case-control portion of the study, there were 500 cases and 480 controls. Hemoglobin data from both DCM and HemoCue methods were accessible for 87 cases and 178 controls at baseline, as well as for 99 cases and 144 controls at the endline. In total, 846 Hb assessments from venous blood were performed using both methods.

The median (IQR) age of children at the time of blood collection was 13.00 (7.00, 15.00) months, out of 846 sample, 420 (49.6%) were from girls. In terms of growth metrics, the mean (SD) length-for-age z-score (LAZ) was −2.70 (0.87) in the case group, −0.99 (0.84) in the control group, and −1.53 (0.99) in the birth cohort group. The mean weight-for-age z-score (WAZ) was −2.64 (0.68) in the case group and −0.50 (0.68) in the control group, with the cohort group having a mean WAZ of −1.13 (1.05). The mean weight-for-length z-score (WLZ) was −0.40 (1.06) for the birth cohort, −1.63 (0.83) for the case group, and 0.03 (0.77) for the control group (Table 1).

**Table 1:** Characteristics of participants in different cohorts including average anthropometry and hemoglobin levels.

Indicator	Case	Control	Cohort	Overall
n	148	230	211	589
Number of samples	186	322	338	846
Age (month), Median (IQR)	14.00 (11.00, 18.00)	13.00 (9.00, 17.00)	8.00 (7.00, 15.00)	13.00 (7.00, 15.00)
Sex, n (%) (Girl)	96 (51.6%)	155 (48.1%)	169 (50.0%)	420 (49.6%)
Length-for-age z score, Mean (SD)	−2.70 (0.87)	−0.99 (0.84)	−1.53 (0.99)	
Weight-for-age z score, Mean (SD)	−2.64 (0.68)	−0.50 (0.68)	−1.13 (1.05)	
Weight-for-length z score, Mean (SD)	−1.63 (0.82)	0.03 (0.77)	−0.40 (1.06)	
Hemoglobin DCM g/dl, Median (IQR)	10.80 (10.30, 11.50)	11.00 (10.30, 11.80)	10.50 (9.80, 11.30)	10.80 (10.10, 11.50)
Hemoglobin HemoCue g/dl, Median (IQR)	11.60 (10.60, 12.50)	12.10 (11.00, 12.80)	11.20 (10.30, 12.10)	11.60 (10.50, 12.50)
Anemia (DCM), n (%) (Yes)	59 (31.7%)	92 (28.6%)	147 (43.5%)	298 (35.2%)
Anemia (HemoCue), n (%) (Yes)	42 (22.6%)	50 (15.5%)	104 (30.8%)	196 (23.2%)

Abbreviations: IQR- Inter quartile range, SD- Standard Deviation, DCM- direct cyanmethemoglobin method.

Note: Number of samples were considering all timepoint.

In the case group, median (IQR) Hb measured using the DCM method was 10.80 (10.30, 11.50) g/dL, compared to 11.00 (10.30, 11.80) g/dL in the control group, and 10.50 (9.80, 11.30) g/dL in the birth cohort group. With a cut-off point <10.5 g/dl for anemia, anemia prevalence was 31.7% of the case group, 28.6% of the control group, and 43.5% of the birth cohort group. Using the HemoCue method, the mean Hb was 11.60 (10.60, 12.50) g/dL for the case group, 12.10 (11.00, 12.80) g/dL for the control group, and 11.20 (10.30, 12.10) g/dL for the birth cohort. Anemia was diagnosed in 22.6% in the case group, 15.5% in the control group, and 30.8% in the birth cohort group (Table 1).

By pooling data from all groups and repeated measurements, the median (IQR) Hb concentration measured by the DCM method was 10.8 (10.1, 11.5) g/dL, and 35.2% of children were classified as anemic. Using the HemoCue method, the median (IQR) Hb concentration was 11.6 (10.5, 12.5) g/dL, with 23.2% of children diagnosed with anemia (S4 Table). The difference in anemia prevalence between the two methods was statistically significant (McNemar's test, p < 0.001).

We observed a strong correlation between the DCM and HemoCue methods, with a Pearson’s correlation coefficient of 0.72. McNemar’s test revealed a statistically significant difference between the methods (p < 0.001), and the Kappa statistic (79.20% agreement, kappa = 0.51, p < 0.001) indicated moderate agreement between the two methods.

To further assess agreement between the DCM and HemoCue methods, we constructed a Bland-Altman plot, where the difference and average of the two methods were calculated for each observation. The plot showed that the average difference (i.e., bias) was close to zero, and more than 95% of the data fell between the lower and upper limits of agreement (Fig 2, S1 Text). Both linear and quadratic regression models were evaluated to assess whether a correction factor could be derived between HemoCue and DCM measurements (S5 Table). However, substantial dispersion in HemoCue measurements and failure to satisfy the linearity assumption prevented development of a reliable correction model. To visually assess linearity and dispersion, DCM hemoglobin values were grouped according to their nearest whole-number category. This categorization was used solely for graphical assessment and did not affect the primary analyses, which were conducted using the original hemoglobin values reported to one decimal place (S2 Fig). Although the agreement between the DCM and HemoCue methods was moderate to strong, our data did not allow us to propose a correction factor.

**Fig 2:**
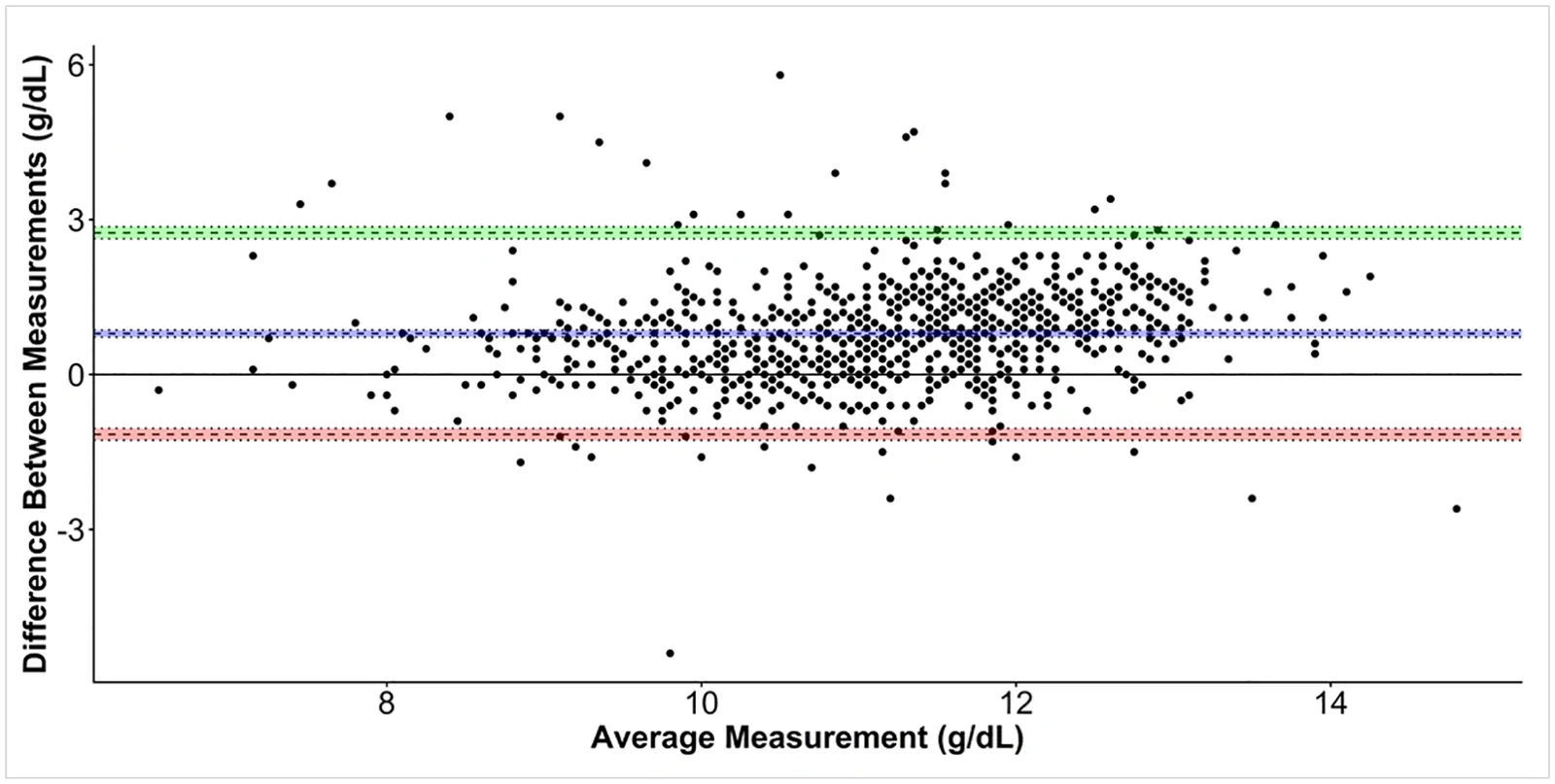
Bland-Altman plot for comparing DCM and HemoCue method. Bland–Altman plot for comparing DCM and HemoCue method agreement analysis (n = 846). Limits of Agreement are shown as dotted black lines with 95% confidence intervals (between lime and rosy-brown color areas), bias (as dotted black line) with 95% confidence interval (blue area).

We then developed a receiver operating characteristic (ROC) curve (Fig 3) using a Hb cut-off point of 10.50 g/dL to diagnose anemia (Table 2). Using anemia defined by the DCM method (Hb < 10.5 g/dL) as the reference standard, ROC analysis of HemoCue hemoglobin measurements yielded an area under the curve (AUC) of 86.55%, with an accuracy of 79.20%, sensitivity of 53.36%, and specificity of 93.25%. The positive predictive value (PPV) was 81.12%, and the negative predictive value (NPV) was 78.62%. Of the total samples, 159 were true positives, 511 were true negatives, 139 were false negatives, and 37 were false positives (Table 3). The optimal Hb cut-off point of 10.9 g/dL was identified based on Youden’s index (Fig 3A).

**Fig 3:**
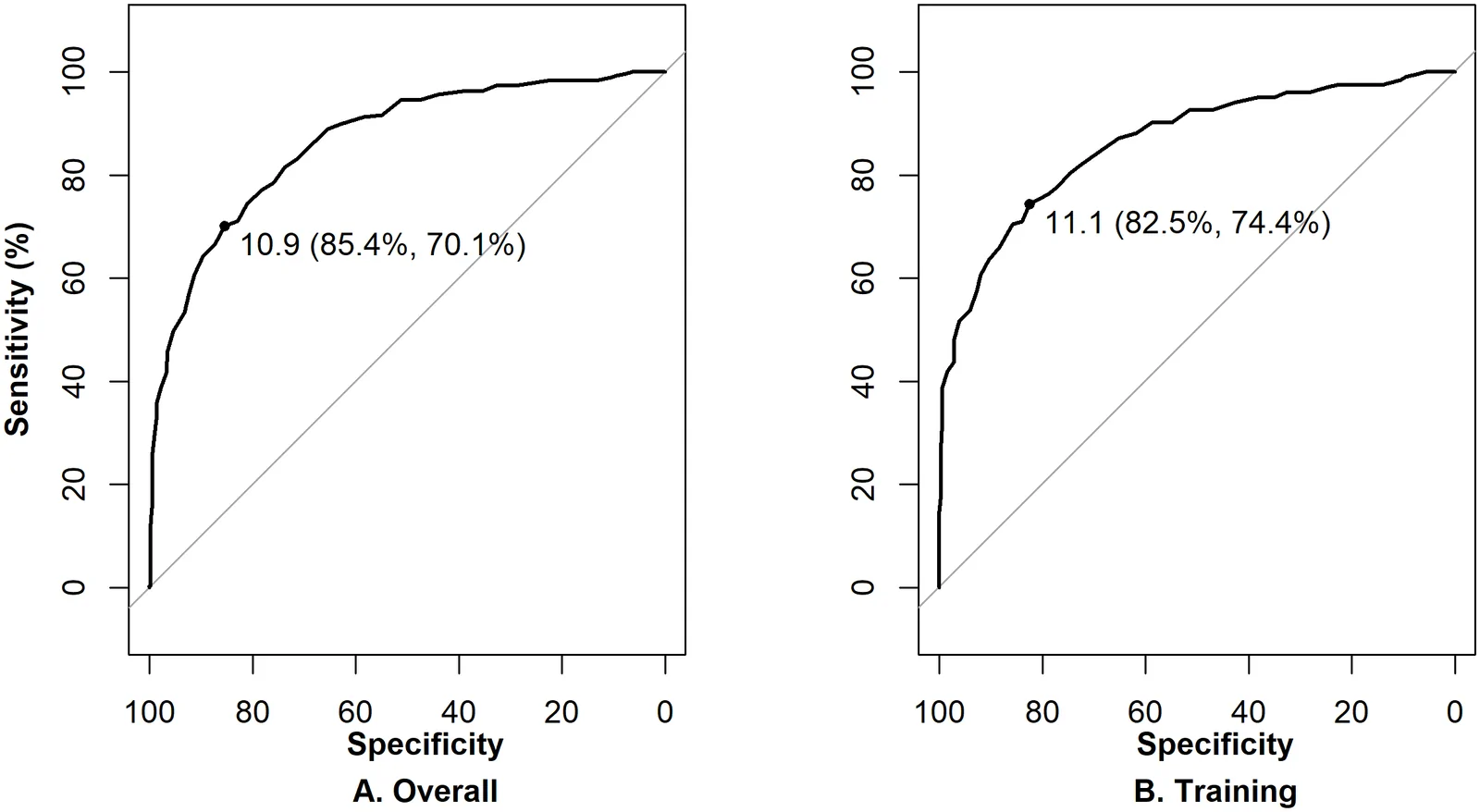
ROC curve with Youden’s index. Youden's index is the value that maximizes the sensitivity and specificity. (A) The optimal hemoglobin cut-off point of 10.9 g/dL was identified based on Youden’s index. (B) The cut-off point of 11.1 g/dL was confirmed in the training set using Youden’s index.

**Table 2:** Diagnostic performance of candidate HemoCue hemoglobin cut-offs identified in the training dataset and subsequently evaluated through internal validation.

ROC analysis	All Hb data	Training data
n	846	591
Area under ROC curve	86.55%	86.26%
Accuracy	79.20%	80.20%
95% CI	76.30%, 81.88%	76.76%, 83.34%
Sensitivity	53.36%	53.69%
Specificity	93.25%	94.07%
Positive predictive value	81.12%	82.58%
Negative predictive value	78.62%	79.52%
True positive	159	109
False negative	139	94
False positive	37	23
True negative	511	365

Abbreviations: Receiver-operating characteristic curve (ROC).

**Table 3:** Different hemoglobin cut-off metric age 6 to 24 month.

Metric	Cut-point 10.80	Cut-point 10.90	Cut-point 11.00	Cut-point 11.10
Accuracy	80.73%	80.02%	80.02%	78.72%
Kappa	0.561	0.552	0.559	0.537
Mcnemar’s Test (p-value)	<0.001	0.021	0.538	0.602
Precision	77.11%	74.16%	72.32%	69.28%
Sensitivity (True Positive Rate)	64.43%	66.44%	70.13%	71.14%
Specificity (True Negative Rate)	89.60%	87.41%	85.40%	82.85%
Positives predicted value	77.11%	74.16%	72.32%	69.28%
Negatives predicted value	82.24%	82.73%	84.02%	84.07%
Detection Rate	22.70%	23.40%	24.70%	25.06%
Balanced Accuracy	77.01%	76.93%	77.77%	76.99%

A threshold value of 11.00 achieved the most favorable trade-off between sensitivity (70.13%) and specificity (85.40%), resulting in the greatest balanced accuracy (77.77%) across the examined cut-offs. At this point, precision was 72.32%, and the kappa statistic (0.559) reflected moderate concordance with the reference standard. In addition, McNemar’s test did not indicate a statistically significant disagreement, further supporting the stability and reliability of this threshold (Table 3).

Next, we validated the new cut-off of 11.00 g/dL through a split-sample analysis. Seventy percent of the data were randomly allocated to a training set and 30% to a test set. In the training set, the AUC was 86.26%, with a sensitivity of 53.69% and a specificity of 94.07% (Table 2).

For the test set, ROC analysis showed an AUC of 86.77%, with a sensitivity of 52.63% and a specificity of 91.25%. After applying the new cut-off of 11.00 g/dL to the test set, the sensitivity has improved to 69.47% (Table 4). Using this new cut-off, 34.2% of children were classified as anemic, compared to 23.2% with the current cut-off of 10.5 g/dL (Table 5 and S6 Table).

**Table 4:** Summary results of ROC curve for test dataset using both the cutoff points.

	Cut-off Hb 10.5 g/dL	Cut-off 11.0 g/dL
The area under ROC curve	86.77%	100.0%
Accuracy	76.86%	78.82%
95% CI	71.19%, 81.90%	73.29%, 83.67%
Sensitivity	52.63%	69.47%
Specificity	91.25%	84.38%
Positive predictive value	78.12%	72.53%
Negative predictive value	76.44%	82.32%
True positive	50	66
False-negative	45	29
False positive	14	25
True negative	146	135

Abbreviations: Receiver-operating characteristic curve (ROC).

**Table 5:** Anemia detection in test dataset with new and old cut-offs.

Anemia detection methods and cut-off (n = 255)	%
Anemic using DCM (cut-off 10.5 g/dL)	35.2
Anemic using HemoCue (cut-off 10.5 g/dL)	23.2
Anemic using HemoCue (cut-off 11.00 g/dL)	34.2

Abbreviations: DCM- direct cyanmethemoglobin method.

Exploratory subgroup analyses were conducted across six predefined groups. None of these analyses were adequately powered. While most of the point estimates suggested cut-off 11.00 g/dL, statistical reliability was limited. Detailed subgroup tables are provided in the Supplementary Materials (S7-S24 Tables). These results should be interpreted cautiously and considered hypothesis generating rather than confirmatory.

## Discussion

Identification of anemia at the population level remains a challenge due to limited diagnostic infrastructure. Children under two years old are vulnerable to anemia and the accurate measurement of Hb is limited in resource poor settings [10]. In this study, we compared the HemoCue method to the gold standard DCM method. Our findings revealed significant discrepancies between the two methods, which can influence the reported prevalence of anemia and subsequent public health decisions.

We compared the Hb levels of the children aged under two years enrolled in the MAL-ED study, using both the DCM and HemoCue methods. The findings highlight important differences between the two measurement methods, which may influence estimates of anemia prevalence and subsequent public health decision-making. The observed variability between HemoCue and DCM measurements suggests that caution is warranted when interpreting hemoglobin values obtained using portable devices and supports the need for context-specific evaluation of diagnostic thresholds. These findings suggest that reliance on HemoCue measurements may lead to systematic differences in anemia classification when compared with reference laboratory methods. Similar variability has been reported in previous studies, highlighting the importance of validating diagnostic thresholds within specific populations and settings [14,22,35]. The Bland-Altman plot demonstrated moderate agreement between the two methods, with more than 95% of observations falling within the limits of agreement. Although the HemoCue method tended to overestimate hemoglobin concentrations relative to the DCM method, the observed bias remained relatively small [36]. These limits were derived from the Bland–Altman analysis and were not evaluated against published total allowable error specifications for hemoglobin. Although analytical precision of the DCM assay was confirmed through quality-control procedures, the limits of agreement observed in the Bland–Altman analysis were not formally evaluated against published total allowable error specifications for hemoglobin. Therefore, a formal assessment of total analytical error could not be performed. In addition, quality-control assessments demonstrated acceptable analytical performance of the DCM assay, with low within-day and between-day imprecision and recovery values close to the assigned target concentrations. Although we attempted to develop a correction factor through polynomial regression, the lack of linearity prevented its development. Then we explored various methods to understand why linearity was not achieved, including stratification by age and Hb levels. When Hb values measured by the DCM method were rounded to the nearest whole number, we observed a considerable dispersion in Hb levels measured by the HemoCue method (S2 Table, S1 and S2 Figs). This wide variation in HemoCue measurements likely accounts for the failure to achieve linearity in the analysis. Despite a moderate to strong agreement between the methods, no correction factor could be proposed based on our data. Therefore, our analysis led to the identification of a new optimal cut-off value of 11.00 g/dL, to better align the HemoCue method with DCM findings. Validation using the training-test approach showed that the new cut-off improved both sensitivity and specificity, with the test dataset achieving 69.47% sensitivity and 84.38% specificity, a marked improvement over the previous cut-off of 10.50 g/dL. Applying this updated cut-off increased the anemia prevalence from 23.2% to 34.2%. This result better aligns with the DCM classification, suggesting a more accurate reflection of anemia burden in this population.

Other studies comparing the HemoCue with automated hematology analyzers, such as the XT1800i (Sysmex) and BC-3000Plus (Mindray), have similarly reported poor agreement. These discrepancies resulted in significant differences in estimated anemia prevalence [16,36]. Other studies comparing HemoCue with reference laboratory methods have reported varying levels of agreement. For example, a study conducted in Ghana found no significant differences in hemoglobin measurements obtained using the HemoCue, cyanmethemoglobin, and Sysmex KX21N methods [37].

Although subgroup analyses can provide insights into potential heterogeneity of effects, the present study was not designed or powered to detect meaningful differences across the six subgroups we examined. These exploratory results highlight areas for future investigation but should not be taken as evidence of definitive subgroup effects. Larger, adequately powered studies are needed to clarify whether the patterns observed here represent true biological or clinical differences.

The minimum required sample size for estimating diagnostic performance with the specified precision was 544 observations. Because the study included 846 paired hemoglobin measurements, the available sample exceeded this requirement, supporting the precision and stability of the estimated diagnostic performance measures. Therefore, the available sample size was sufficient to provide precise estimates of diagnostic performance.

The strength of this study is in its use of venous blood samples, which allowed us to conduct direct comparisons of the two methods under controlled conditions. This approach minimizes biological variability often introduced by differences in blood sample type (e.g., capillary, venous, or arterial), as reported in earlier studies [38–40]. A recent study suggested that accurate and precise determination of Hb requires venous blood. The use of single-drop capillary blood is not recommended due to its high measurement variability [41]. However, a study in Hong Kong reported, the capillary point of care test (POCT HemoCue) had acceptable agreement with venous Hb in the emergency department setting [42]. Furthermore, our use of samples from a well-regulated study, including repeated measurements over time, and regular quality controls for Hemocue analyzers, enabled a robust comparison and validation of the new cut-off value. The train-test validation process enhanced the robustness of the proposed cut-off by providing internal validation within the study dataset. However, because both datasets originated from the same study population, external validation in independent populations remains necessary before broader implementation [43].

Despite these strengths, our study has a few limitations. While venous blood sampling ensures greater accuracy, it is not the standard collection method in field settings where HemoCue is often used, particularly in resource-poor areas. The validity of our findings for capillary blood samples, which are commonly used in field surveys and community-based screening programs, remains unverified. Because paired capillary and venous measurements were not available in this study, we could not evaluate whether the proposed HemoCue cut-off of 11.0 g/dL performs similarly under field conditions. Furthermore, we perform subgroup analysis to assess whether the performance of the proposed cut-off varied according to age, nutritional status (WAZ or LAZ), or inflammatory status. None of these analyses were adequately powered. These results should be interpreted cautiously and considered hypothesis generating rather than confirmatory. Future studies should validate this threshold using paired capillary and venous samples and explore potential subgroup-specific differences in diagnostic performance. Previous research has found significant variability in Hb levels depending on the site of blood collection. Future studies should therefore examine whether the proposed cut-off holds. Our study focused exclusively on children aged under two years of age in Bangladesh. This cohort provides valuable insights into the performance of the HemoCue method in a low-resource setting, the findings may not be generalizable to other regions or populations. Factors such as altitude, nutritional status, and the prevalence of genetic hemoglobinopathies could influence Hb levels and affect the applicability of this cut-off [44–46]. Validation studies across diverse geographic and demographic settings are therefore essential to ensure broader applicability. While we identified a new cut-off value to improve anemia classification using HemoCue, the inherent limitations of the device, such as its tendency to overestimate Hb persist. Efforts to improve the accuracy of portable hemoglobinometers like HemoCue should continue, as they are indispensable in large-scale surveys and fieldwork where access to laboratory-based methods is not feasible. Our analyses included repeated measurements from some children, which may introduce within-child correlation and challenge the assumption of independence, we do not believe this significantly affects the robustness or interpretability of our findings. Although the primary objective of this study was method comparison rather than longitudinal modeling, future studies could apply mixed-effects or generalized estimating equation approaches to explicitly account for intra-child correlation and evaluate its impact on diagnostic performance estimates.

Our findings are specific to the HemoCue 201 model used in this study. Previous studies have documented differences in analytical performance between HemoCue models, including the HemoCue 201 and HemoCue 301. Therefore, the proposed 11.0 g/dL cut-off should not be assumed to apply to other HemoCue devices without further validation [47].

Adopting a corrected cut-off of 11.00 g/dL for HemoCue use in low-resource settings would directly address the issue of systematic Hb overestimation and improve the identification of anemic children. Despite its limitations, the HemoCue 201 device continues to be used in many clinical and field settings because of its portability and operational simplicity. Our findings suggest that careful consideration of diagnostic thresholds may improve the accuracy of anemia classification when this device is used. By applying the revised cut-off value of 11.00 g/dL, health programs can more accurate estimates of anemia prevalence and reduce the risk of missing children who require treatment.

## Conclusion

Our comparison between the HemoCue and DCM methods for measuring Hb levels and detecting anemia shows that HemoCue tends to overestimate Hb levels. By proposing and validating a new cut-off value for the HemoCue method, we provide a more accurate way of diagnosing anemia in children aged under two years. In settings where the HemoCue 201 device is used for hemoglobin assessment, careful consideration of diagnostic thresholds may improve the accuracy of anemia classification and population-level estimates of anemia burden. Future research should focus on further validating this cut-off value across diverse populations and blood types. This will help ensure that the true burden of anemia is correctly identified and properly addressed in public health programs initiatives.

## Supporting information

S1 Text Detailed results from Bland-Altman plot for comparing DCM and HemoCue method. (DOCX)

S1 Table Participant baseline characteristics across birth cohorts by availability status. (DOCX)

S2 Table Participant baseline characteristics across case group by availability status. (DOCX)

S3 Table Participant baseline characteristics across control group by availability status. (DOCX)

S4 Table Comparison of hemoglobin concentration using direct cyanmethemoglobin and HemoCue methods in children enrolled in Mal-ED studies, Bangladesh. (DOCX)

S5 Table Polynomial regression. (DOCX)

S6 Table 2 × 2 contingency table using complete dataset (n = 846). (DOCX)

S7 Table Infant (age 6–12 months) participants baseline characteristics. (DOCX)

S8 Table Different hemoglobin cut-off metric for infant (age 6–12 months). (DOCX)

S9 Table Anemia detection in test dataset with new and old cut-offs for infant (age 6–12 months). (DOCX)

S10 Table Toddler (age 12–24 months) participants baseline characteristics. (DOCX)

S11 Table Different hemoglobin cut-off metric for toddler (age 12–24 months). (DOCX)

S12 Table Anemia detection in test dataset with new and old cut-offs for toddler (age 12–24 months). (DOCX)

S13 Table Stunted (length-for-age ≤ –2 SD) participants baseline characteristics. (DOCX)

S14 Table Different hemoglobin cut-off metric for stunted (length-for-age ≤ –2 SD) children. (DOCX)

S15 Table Anemia detection in test dataset with new and old cut-offs for stunted (length-for-age ≤ –2 SD) children. (DOCX)

S16 Table Underweight (weight-for-age ≤ –2 SD) participants baseline characteristics. (DOCX)

S17 Table Different hemoglobin cut-off metric for underweight (weight-for-age ≤ –2 SD) children. (DOCX)

S18 Table Anemia detection in test dataset with new and old cut-offs for underweight (weight-for-age ≤ –2 SD) children. (DOCX)

S19 Table Participants baseline characteristics based on acute systemic inflammation status using C-reactive protein (CRP) cut-off of ≥ 5 mg/L. (DOCX)

S20 Table Different hemoglobin cut-off metric for acute systemic inflammation status using C-reactive protein (CRP) cut-off of ≥ 5 mg/L. (DOCX)

S21 Table Anemia detection in test dataset with new and old cut-offs for acute systemic inflammation status using C-reactive protein (CRP) cut-off of ≥ 5 mg/L. (DOCX)

S22 Table Participants baseline characteristics based on acute systemic inflammation status using Alpha-1-Acid Glycoprotein (AGP) cut-off of ≥ 100 mg/dL. (DOCX)

S23 Table Different hemoglobin cut-off metric for acute systemic inflammation status using Alpha-1-Acid Glycoprotein (AGP) cut-off of ≥ 100 mg/dL. (DOCX)

S24 Table Anemia detection in test dataset with new and old cut-offs for acute systemic inflammation status using Alpha-1-Acid Glycoprotein (AGP) cut-off of ≥ 100 mg/dL. (DOCX)

S1 Fig Polynomial regression analysis to test linearity. To suggest a correction factor, a simple polynomial regression of degree 2 (quadratic model) was performed. However, the assumption of linearity was not satisfied. Subsequently, paired hemoglobin concentrations measured were excluded if hemoglobin concentrations using the direct cyanmethemoglobin method were less than 7 or greater than 14, approximately 2% of the data. Despite this adjustment, the linear model still failed to achieve linearity.
(TIF)

S2 Fig Scatter plot to assess of linearity by stratifying hemoglobin levels measured by DCM. A scatter plot was created to analyze hemoglobin concentrations using both the direct cyanmethemoglobin method and the HemoCue method. The plot was stratified by rounding the direct cyanmethemoglobin values to the nearest whole number, allowing for an assessment of the distribution of hemoglobin concentrations measured by the HemoCue method.
(TIF)
