# Supplementary material for: Validation of HemoCue method and development of a revised hemoglobin cut-off to detect anemia in children aged 6–24-months
Source: PLoS One. 2026 Oct 16;21(10):e0346506. doi: 10.1371/journal.pone.0346506 (PMC13674700; doi:10.1371/journal.pone.0346506)
Supplement: Supplementary file 1 [file pone.0346506.s001.docx]

**S1 Text. Detailed results from Bland-Altman plot for comparing DCM and HemoCue method**

| Number of comparisons: 846  Maximum value for average measures: 14.8  Minimum value for average measures: 6.55  Maximum value for difference in measures: 5.8  Minimum value for difference in measures: -5.4  Derived measures:  Mean of differences/means: 7.019665  Point estimate of bias as proportion of lowest average: 12.10907  Point estimate of bias as proportion of highest average 5.359082  Spread of data between lower and upper LoAs: 3.904103  Bias as proportion of LoA spread: 20.31566 | Bias: 0.7931442  Standard deviation of bias: 0.9959446  Standard error of bias: 0.03424128  Standard error for limits of agreement: 0.0585416  Bias- upper 95% CI: 0.8603521  Bias- lower 95% CI: 0.7259363  Upper limit of agreement: 2.745196  Upper LOA- upper 95% CI: 2.8601  Upper LOA- lower 95% CI: 2.630292  Lower limit of agreement: -1.158907  Lower LOA- upper 95% CI: -1.044003  Lower LOA- lower 95% CI: -1.273811 |
| --- | --- |
