# Supplementary material for: Validation of HemoCue method and development of a revised hemoglobin cut-off to detect anemia in children aged 6–24-months
Source: PLoS One. 2026 Oct 16;21(10):e0346506. doi: 10.1371/journal.pone.0346506 (PMC13674700; doi:10.1371/journal.pone.0346506)
Supplement: Supplementary file 2 [file pone.0346506.s002.docx]

**S1 Table. Participant baseline characteristics across birth cohorts by availability status**

| Characteristic | n | Overall  n = 240 | Without measurements  n = 29 | With measurements  n = 211 | p-value |
| --- | --- | --- | --- | --- | --- |
| Age (month), Median (IQR) | 240 | 7.0 (6.9, 7.0) | 7.0 (6.9, 7.0) | 7.0 (6.9, 7.0) | 0.969^1^ |
| Girls, n (%) | 240 | 123 (51%) | 14 (48%) | 109 (52%) | 0.733^2^ |
| Weight (kg), Median (IQR) | 233 | 7.1 (6.6, 7.7) | 7.1 (6.2, 7.3) | 7.1 (6.6, 7.7) | 0.244^1^ |
| Height (cm), Median (IQR) | 233 | 65.5 (64.0, 66.7) | 65.1 (62.8, 66.5) | 65.5 (64.0, 66.7) | 0.322^1^ |
| Weight-for-age z-score, Mean (SD) | 233 | -1.0 (1.1) | -1.4 (1.1) | -0.9 (1.1) | 0.053^3^ |
| Length/height-for-age z-score, Mean (SD) | 232 | -1.3 (1.0) | -1.5 (0.8) | -1.2 (1.0) | 0.207^3^ |
| Weight-for-length/height z-score, Mean (SD) | 233 | -0.2 (1.0) | -0.4 (0.7) | -0.2 (1.1) | 0.155^3^ |
| Mother's age, Median (IQR) | 240 | 25.0 (21.0, 28.0) | 25.0 (22.0, 28.0) | 25.0 (21.0, 28.0) | 0.441^1^ |
| Mother's complete years of education, Median (IQR) | 240 | 5.0 (2.0, 7.0) | 5.0 (2.0, 8.0) | 5.0 (2.0, 7.0) | 0.281^1^ |
| Household head age, Median (IQR) | 235 | 35.0 (30.0, 45.0) | 35.0 (30.0, 40.0) | 35.0 (30.0, 46.0) | 0.245^1^ |
| Female headed household, n (%) | 235 | 12 (5.1%) | 3 (10%) | 9 (4.4%) | 0.173^4^ |
| Household's head complete years of education, Median (IQR) | 235 | 4.0 (0.0, 7.0) | 5.0 (0.0, 9.0) | 3.0 (0.0, 7.0) | 0.298^1^ |
| People per room, Median (IQR) | 240 | 3.3 (3.0, 4.0) | 3.0 (3.0, 4.0) | 3.5 (3.0, 4.0) | 0.255^1^ |
| Handwashing with soap after helping child defecate, n (%) | 240 | 175 (73%) | 22 (76%) | 153 (73%) | 0.703^2^ |
| Handwashing with soap before preparing food, n (%) | 240 | 55 (23%) | 8 (28%) | 47 (22%) | 0.523^2^ |
| Handwashing with soap after using toilet, n (%) | 240 | 182 (76%) | 21 (72%) | 161 (76%) | 0.646^2^ |
| Improved drinking water source (WHO), n (%) | 240 | 240 (100%) | 29 (100%) | 211 (100%) | >0.999^4^ |
| Access to improved toilet system (WHO), n (%) | 240 | 27 (11%) | 5 (17%) | 22 (10%) | 0.341^4^ |
| Toilet shared with other family, n (%) | 240 | 200 (83%) | 24 (83%) | 176 (83%) | >0.999^4^ |
| Average monthly household income, Median (IQR) | 240 | 8,000 (6,000, 10,000) | 7,000 (6,000, 12,000) | 8,000 (6,000, 10,000) | 0.999^1^ |
| Abbreviations: IQR- Inter quartile range, SD- Standard Deviation  p-value obtained from ^1^Wilcoxon rank sum test, ^2^Pearson's Chi-squared test, ^3^Welch Two Sample t-test, ^4^Fisher's exact test | | | | | |
