# Supplementary material for: Validation of HemoCue method and development of a revised hemoglobin cut-off to detect anemia in children aged 6–24-months
Source: PLoS One. 2026 Oct 16;21(10):e0346506. doi: 10.1371/journal.pone.0346506 (PMC13674700; doi:10.1371/journal.pone.0346506)
Supplement: Supplementary file 3 [file pone.0346506.s003.docx]

**S2 Table. Participant baseline characteristics across case group by availability status**

| Characteristic | n | Overall  n = 500 | Without measurements  n = 352 | With measurements  n = 148 | p-value |
| --- | --- | --- | --- | --- | --- |
| Age (month), Median (IQR) | 500 | 13.9 (9.4, 18.7) | 15.3 (10.5, 19.6) | 11.2 (8.3, 15.5) | <0.001^1^ |
| Girls, n (%) | 500 | 242 (48%) | 166 (47%) | 76 (51%) | 0.392^2^ |
| Weight (kg), Median (IQR) | 500 | 7.1 (6.4, 8.0) | 7.3 (6.5, 8.2) | 6.6 (6.1, 7.4) | <0.001^1^ |
| Height (cm), Median (IQR) | 499 | 70.2 (66.0, 74.6) | 71.7 (67.0, 75.6) | 68.0 (64.4, 72.5) | <0.001^1^ |
| Weight-for-age z-score, Mean (SD) | 500 | -2.7 (0.6) | -2.7 (0.5) | -2.8 (0.7) | 0.074^3^ |
| Length/height-for-age z-score, Mean (SD) | 499 | -2.6 (0.9) | -2.5 (0.9) | -2.6 (0.9) | 0.962^3^ |
| Weight-for-length/height z-score, Mean (SD) | 498 | -1.8 (0.7) | -1.8 (0.7) | -1.9 (0.8) | 0.420^3^ |
| Mother's age, Median (IQR) | 500 | 23.0 (21.0, 27.5) | 23.0 (21.0, 27.5) | 24.0 (21.0, 27.5) | 0.872^1^ |
| Mother's complete years of education, Median (IQR) | 500 | 4.0 (0.0, 6.0) | 4.0 (1.0, 6.0) | 4.0 (0.0, 6.0) | 0.694^1^ |
| Household head age, Median (IQR) | 490 | 33.0 (28.0, 40.0) | 33.0 (28.0, 40.0) | 33.0 (26.0, 42.0) | 0.785^1^ |
| Female headed household, n (%) | 490 | 41 (8.4%) | 29 (8.4%) | 12 (8.3%) | 0.962^2^ |
| Household's head complete years of education, Median (IQR) | 490 | 4.0 (0.0, 7.0) | 4.0 (0.0, 6.0) | 5.0 (0.0, 7.0) | 0.250^1^ |
| People per room, Median (IQR) | 500 | 3.5 (3.0, 4.0) | 3.5 (3.0, 4.0) | 3.5 (3.0, 4.5) | 0.769^1^ |
| Handwashing with soap after helping child defecate, n (%) | 500 | 307 (61%) | 214 (61%) | 93 (63%) | 0.668^2^ |
| Handwashing with soap before preparing food, n (%) | 499 | 113 (23%) | 71 (20%) | 42 (29%) | 0.041^2^ |
| Handwashing with soap after using toilet, n (%) | 500 | 365 (73%) | 260 (74%) | 105 (71%) | 0.502^2^ |
| Improved drinking water source (WHO), n (%) | 500 | 500 (100%) | 352 (100%) | 148 (100%) | >0.999^4^ |
| Access to improved toilet system (WHO), n (%) | 500 | 27 (5.4%) | 22 (6.3%) | 5 (3.4%) | 0.195^2^ |
| Toilet shared with other family, n (%) | 500 | 459 (92%) | 321 (91%) | 138 (93%) | 0.446^2^ |
| Average monthly household income, Median (IQR) | 500 | 7,000 (5,000, 9,000) | 7,000 (5,000, 9,000) | 7,000 (5,000, 10,000) | 0.548^1^ |
| Abbreviations: IQR- Inter quartile range, SD- Standard Deviation  p-value obtained from ^1^Wilcoxon rank sum test, ^2^Pearson's Chi-squared test, ^3^Welch Two Sample t-test, ^4^Fisher's exact test | | | | | |
