# Supplementary material for: Validation of HemoCue method and development of a revised hemoglobin cut-off to detect anemia in children aged 6–24-months
Source: PLoS One. 2026 Oct 16;21(10):e0346506. doi: 10.1371/journal.pone.0346506 (PMC13674700; doi:10.1371/journal.pone.0346506)
Supplement: Supplementary file 4 [file pone.0346506.s004.docx]

**S3 Table. Participant baseline characteristics across control group by availability status**

| Characteristic | n | Overall  n = 480 | Without measurements  n = 250 | With measurements  n = 230 | p-value |
| --- | --- | --- | --- | --- | --- |
| Age (month), Median (IQR) | 480 | 11.0 (7.5, 15.5) | 12.0 (8.2, 16.3) | 9.9 (7.3, 14.3) | 0.001^1^ |
| Girls, n (%) | 480 | 232 (48%) | 122 (49%) | 110 (48%) | 0.831^2^ |
| Weight (kg), Median (IQR) | 480 | 8.7 (7.9, 9.7) | 8.8 (8.1, 9.9) | 8.5 (7.8, 9.4) | 0.004^1^ |
| Height (cm), Median (IQR) | 478 | 71.6 (67.8, 76.9) | 73.4 (68.5, 78.0) | 71.0 (67.3, 75.0) | <0.001^1^ |
| Weight-for-age z-score, Mean (SD) | 480 | -0.4 (0.5) | -0.4 (0.4) | -0.4 (0.5) | 0.526^3^ |
| Length/height-for-age z-score, Mean (SD) | 478 | -0.7 (0.8) | -0.7 (0.8) | -0.8 (0.8) | 0.211^3^ |
| Weight-for-length/height z-score, Mean (SD) | 478 | 0.0 (0.6) | 0.0 (0.6) | 0.1 (0.7) | 0.038^3^ |
| Mother's age, Median (IQR) | 480 | 25.0 (22.0, 28.0) | 25.0 (21.0, 28.0) | 24.0 (22.0, 28.0) | 0.766^1^ |
| Mother's complete years of education, Median (IQR) | 480 | 5.0 (3.0, 8.0) | 5.0 (2.0, 8.0) | 5.0 (3.0, 8.0) | 0.207^1^ |
| Household head age, Median (IQR) | 469 | 34.0 (28.0, 40.0) | 35.0 (30.0, 40.0) | 32.0 (28.0, 38.0) | 0.012^1^ |
| Female headed household, n (%) | 469 | 32 (6.8%) | 19 (7.8%) | 13 (5.8%) | 0.389^2^ |
| Household's head complete years of education, Median (IQR) | 469 | 5.0 (1.0, 9.0) | 5.0 (1.0, 8.0) | 5.0 (2.0, 9.0) | 0.210^1^ |
| People per room, Median (IQR) | 480 | 3.0 (3.0, 4.0) | 3.0 (3.0, 4.0) | 3.0 (3.0, 4.0) | 0.604^1^ |
| Handwashing with soap after helping child defecate, n (%) | 480 | 328 (68%) | 175 (70%) | 153 (67%) | 0.413^2^ |
| Handwashing with soap before preparing food, n (%) | 480 | 116 (24%) | 58 (23%) | 58 (25%) | 0.606^2^ |
| Handwashing with soap after using toilet, n (%) | 480 | 380 (79%) | 191 (76%) | 189 (82%) | 0.120^2^ |
| Improved drinking water source (WHO), n (%) | 480 | 480 (100%) | 250 (100%) | 230 (100%) | >0.999^4^ |
| Access to improved toilet system (WHO), n (%) | 480 | 64 (13%) | 36 (14%) | 28 (12%) | 0.474^2^ |
| Toilet shared with other family, n (%) | 480 | 388 (81%) | 198 (79%) | 190 (83%) | 0.343^2^ |
| Average monthly household income, Median (IQR) | 480 | 8,000 (6,000, 12,000) | 8,000 (6,000, 12,000) | 9,000 (6,000, 12,000) | 0.189^1^ |
| Abbreviations: IQR- Inter quartile range, SD- Standard Deviation  p-value obtained from ^1^Wilcoxon rank sum test, ^2^Pearson's Chi-squared test, ^3^Welch Two Sample t-test, ^4^Fisher's exact test | | | | | |
