# Supplementary material for: Validation of HemoCue method and development of a revised hemoglobin cut-off to detect anemia in children aged 6–24-months
Source: PLoS One. 2026 Oct 16;21(10):e0346506. doi: 10.1371/journal.pone.0346506 (PMC13674700; doi:10.1371/journal.pone.0346506)
Supplement: Supplementary file 5 [file pone.0346506.s005.docx]

**S4 Table. Comparison of hemoglobin concentration using direct cyanmethemoglobin and HemoCue methods in children enrolled in Mal-ED studies, Bangladesh**

| Characteristic | DCM  (n = 846) | HemoCue  (n = 846) |
| --- | --- | --- |
| Age (month), Median (IQR) | 13.0 (7.0, 15.0) | 13.0 (7.0, 15.0) |
| Girls, n (%) | 420 (49.6%) | 420 (49.6%) |
| Hemoglobin in g/dL, Median (IQR) | 10.8 (10.1, 11.5) | 11.6 (10.5, 12.5) |
| Anemia, n (%) | 298 (35.2%) | 196 (23.2%) |
| Abbreviations: IQR- Inter quartile range, DCM- direct cyanmethemoglobin method | | |
