# Supplementary material for: Validation of HemoCue method and development of a revised hemoglobin cut-off to detect anemia in children aged 6–24-months
Source: PLoS One. 2026 Oct 16;21(10):e0346506. doi: 10.1371/journal.pone.0346506 (PMC13674700; doi:10.1371/journal.pone.0346506)
Supplement: Supplementary file 6 [file pone.0346506.s006.docx]

**S5 Table. Polynomial regression**

| Indicator | Coefficient (95% CI) | p-value |
| --- | --- | --- |
| Liner regression |  |  |
| Constant (βo) | 3.58 (3.11, 4.05) | <0.001 |
| HemoCue | 0.621 (0.58, 0.66) | <0.001 |
| Quadratic regression |  |  |
| Constant (βo) | 0.22 (-2.48, 2.93) | 0.872 |
| HemoCue | 1.22 (0.74, 1.70) | <0.001 |
| HemoCue (square) | -0.03 (-0.05, -0.01) | 0.014 |
