# Supplementary material for: Validation of HemoCue method and development of a revised hemoglobin cut-off to detect anemia in children aged 6–24-months
Source: PLoS One. 2026 Oct 16;21(10):e0346506. doi: 10.1371/journal.pone.0346506 (PMC13674700; doi:10.1371/journal.pone.0346506)
Supplement: Supplementary file 7 [file pone.0346506.s007.docx]

**S6 Table. 2 × 2 contingency table using complete dataset (n = 846)**

| Anemic using cut-off 11.00 g/dL | Anemic using DCM  (cut-off 10.5 g/dL) | |
| --- | --- | --- |
|  | No | Yes |
| No | 468 (55.3%) | 89 (10.5%) |
| Yes | 80 (9.4%) | 209 (27.7%) |
|  |  |  |
| Anemic using cut-off 11.00 g/dL | Anemic using HemoCue  (cut-off 10.5 g/dL) | |
|  | No | Yes |
| No | 557 (65.8%) | 0 (0.0%) |
| Yes | 93 (11.0%) | 196 (23.2%) |

Abbreviations: DCM- direct cyanmethemoglobin method
