# Supplementary material for: Validation of HemoCue method and development of a revised hemoglobin cut-off to detect anemia in children aged 6–24-months
Source: PLoS One. 2026 Oct 16;21(10):e0346506. doi: 10.1371/journal.pone.0346506 (PMC13674700; doi:10.1371/journal.pone.0346506)
Supplement: Supplementary file 8 [file pone.0346506.s008.docx]

**S7 Table. Infant (age 6 to 12 months) participants baseline characteristics**

| Indicator | Case | Control | Cohort | Overall |
| --- | --- | --- | --- | --- |
| Number of samples | 50 | 126 | 173 | 349 |
| Age (month), Median (IQR) | 9.00 (7.00, 11.00) | 8.00 (7.00, 10.00) | 7.00 (7.00, 7.00) | 7.00 (7.00, 8.00) |
| Sex, n (%) (Girl) | 25 (50.0%) | 65 (51.6%) | 82 (47.4%) | 172 (49.3%) |
| Length-for-age z score, Mean (SD) | -2.50 (0.81) | -0.78 (0.86) | -1.27 (0.99) |  |
| Weight-for-age z score, Mean (SD) | -2.66 (0.66) | -0.33 (0.62) | -0.88 (1.05) |  |
| Weight-for-length z score, Mean (SD) | -1.60 (0.70) | 0.18 (0.70) | -0.08 (1.00) |  |
| Hemoglobin HemoCue g/dl, Median (IQR) | 11.05 (10.40, 11.90) | 12.05 (11.10, 12.80) | 11.10 (10.20, 12.00) | 11.40 (10.50, 12.40) |
| Hemoglobin DCM g/dl, Median (IQR) | 10.50 (10.00, 11.00) | 11.00 (10.40, 11.60) | 10.70 (10.10, 11.40) | 10.80 (10.20, 11.40) |
| Anemia (HemoCue), n (%) (Yes) | 15 (30.0%) | 14 (11.1%) | 55 (31.8%) | 84 (24.1%) |
| Anemia (DCM), n (%) (Yes) | 23 (46.0%) | 32 (25.4%) | 63 (36.4%) | 118 (33.8%) |

Abbreviations: IQR- Inter quartile range, SD- Standard Deviation, DCM- direct cyanmethemoglobin method
