# Supplementary material for: Validation of HemoCue method and development of a revised hemoglobin cut-off to detect anemia in children aged 6–24-months
Source: PLoS One. 2026 Oct 16;21(10):e0346506. doi: 10.1371/journal.pone.0346506 (PMC13674700; doi:10.1371/journal.pone.0346506)
Supplement: Supplementary file 9 [file pone.0346506.s009.docx]

**S8 Table. Different hemoglobin cut-off metric for infant (age 6 to 12 months)**

| Metric | Cut-point 10.80 | Cut-point 10.90 | Cut-point 11.00 | Cut-point 11.10 |
| --- | --- | --- | --- | --- |
| Accuracy | 79.37% | 77.65% | 76.50% | 75.07% |
| Kappa | 0.535 | 0.507 | 0.492 | 0.466 |
| Mcnemar’s Test (p-value) | 0.724 | 0.571 | 0.098 | 0.032 |
| Precision | 70.18% | 66.13% | 63.43% | 61.15% |
| Sensitivity (True Positive Rate) | 67.80% | 69.49% | 72.03% | 72.03% |
| Specificity (True Negative Rate) | 85.28% | 81.82% | 78.79% | 76.62% |
| Positives predicted value | 70.18% | 66.13% | 63.43% | 61.15% |
| Negatives predicted value | 83.83% | 84.00% | 84.65% | 84.29% |
| Detection Rate | 22.92% | 23.50% | 24.36% | 24.36% |
| Balanced Accuracy | 76.54% | 75.65% | 75.41% | 74.33% |
