# Supplementary material for: Validation of HemoCue method and development of a revised hemoglobin cut-off to detect anemia in children aged 6–24-months
Source: PLoS One. 2026 Oct 16;21(10):e0346506. doi: 10.1371/journal.pone.0346506 (PMC13674700; doi:10.1371/journal.pone.0346506)
Supplement: Supplementary file 11 [file pone.0346506.s011.docx]

**S10 Table. Toddler (age 12 to 24 months) participants baseline characteristics**

| Indicator | Case | Control | Cohort | Overall |
| --- | --- | --- | --- | --- |
| Number of samples | 136 | 196 | 165 | 497 |
| Age (month), Median (IQR) | 16.00 (14.00, 20.00) | 15.00 (13.00, 19.00) | 15.00 (15.00, 15.00) | 15.00 (14.00, 18.00) |
| Sex, n (%) (Girl) | 71 (52.2%) | 90 (45.9%) | 87 (52.7%) | 248 (49.9%) |
| Length-for-age z score, Mean (SD) | -2.79 (0.88) | -1.14 (0.80) | -1.81 (0.92) |  |
| Weight-for-age z score, Mean (SD) | -2.63 (0.69) | -0.61 (0.69) | -1.39 (0.99) |  |
| Weight-for-length z score, Mean (SD) | -1.64 (0.87) | -0.07 (0.80) | -0.74 (1.01) |  |
| Hemoglobin HemoCue g/dl, Median (IQR) | 11.90 (10.80, 12.70) | 12.10 (11.00, 12.90) | 11.40 (10.30, 12.30) | 11.80 (10.60, 12.70) |
| Hemoglobin DCM g/dl, Median (IQR) | 11.00 (10.40, 11.60) | 11.00 (10.20, 11.90) | 10.40 (9.50, 11.30) | 10.90 (10.00, 11.60) |
| Anemia (HemoCue), n (%) (Yes) | 27 (19.9%) | 36 (18.4%) | 49 (29.7%) | 112 (22.5%) |
| Anemia (DCM), n (%) (Yes) | 36 (26.5%) | 60 (30.6%) | 84 (50.9%) | 180 (36.2%) |

Abbreviations: IQR- Inter quartile range, SD- Standard Deviation, DCM- direct cyanmethemoglobin method
