# Supplementary material for: Validation of HemoCue method and development of a revised hemoglobin cut-off to detect anemia in children aged 6–24-months
Source: PLoS One. 2026 Oct 16;21(10):e0346506. doi: 10.1371/journal.pone.0346506 (PMC13674700; doi:10.1371/journal.pone.0346506)
Supplement: Supplementary file 12 [file pone.0346506.s012.docx]

**S11 Table. Different hemoglobin cut-off metric for toddler (age 12 to 24 months)**

| Metric | Cut-point 10.80 | Cut-point 10.90 | Cut-point 11.00 | Cut-point 11.10 |
| --- | --- | --- | --- | --- |
| Accuracy | 81.69% | 81.69% | 82.49% | 81.29% |
| Kappa | 58.11% | 58.53% | 60.94% | 58.86% |
| Mcnemar’s Test (p-value) | <0.001 | <0.001 | 0.010 | 0.213 |
| Precision | 0.830 | 0.811 | 0.800 | 0.761 |
| Sensitivity (True Positive Rate) | 62.22% | 64.44% | 68.89% | 70.56% |
| Specificity (True Negative Rate) | 92.74% | 91.48% | 90.22% | 87.38% |
| Positives predicted value | 82.96% | 81.12% | 80.00% | 76.05% |
| Negatives predicted value | 81.22% | 81.92% | 83.63% | 83.94% |
| Detection Rate | 22.54% | 23.34% | 24.95% | 25.55% |
| Balanced Accuracy | 77.48% | 77.96% | 79.55% | 78.97% |
