# Supplementary material for: Validation of HemoCue method and development of a revised hemoglobin cut-off to detect anemia in children aged 6–24-months
Source: PLoS One. 2026 Oct 16;21(10):e0346506. doi: 10.1371/journal.pone.0346506 (PMC13674700; doi:10.1371/journal.pone.0346506)
Supplement: Supplementary file 13 [file pone.0346506.s013.docx]

**S12 Table. Anemia detection in test dataset with new and old cut-offs for toddler (age 12 to 24 months)**

| Anemia detection methods and cut-off (n= 150) | **%** |
| --- | --- |
| Anemic using DCM (cut-off 10.5 g/dL) | 37.3 |
| Anemic using HemoCue (cut-off 10.5 g/dL) | 25.3 |
| Anemic using HemoCue (cut-off 11.00 g/dL) | 30.0 |

Abbreviations: DCM- direct cyanmethemoglobin method
