# Supplementary material for: Validation of HemoCue method and development of a revised hemoglobin cut-off to detect anemia in children aged 6–24-months
Source: PLoS One. 2026 Oct 16;21(10):e0346506. doi: 10.1371/journal.pone.0346506 (PMC13674700; doi:10.1371/journal.pone.0346506)
Supplement: Supplementary file 14 [file pone.0346506.s014.docx]

**S13 Table. Stunted (length-for-age ≤ –2 SD) participants baseline characteristics**

| Indicator | Case | Control | Cohort | Overall |
| --- | --- | --- | --- | --- |
| Number of samples | 136 | 33 | 102 | 271 |
| Age (month), Median (IQR) | 14.00 (11.00, 18.50) | 13.00 (12.00, 19.00) | 15.00 (7.00, 15.00) | 15.00 (11.00, 16.00) |
| Sex, n (%) (Girl) | 69 (50.7%) | 16 (48.5%) | 41 (40.2%) | 126 (46.5%) |
| Length-for-age z score, Mean (SD) | -2.98 (0.73) | -2.37 (0.24) | -2.66 (0.62) |  |
| Weight-for-age z score, Mean (SD) | -2.73 (0.68) | -1.19 (0.55) | -1.96 (0.88) |  |
| Weight-for-length z score, Mean (SD) | -1.55 (0.83) | 0.02 (0.81) | -0.71 (1.04) |  |
| Hemoglobin HemoCue g/dl, Median (IQR) | 11.60 (10.45, 12.50) | 11.70 (11.00, 12.40) | 10.90 (10.10, 12.00) | 11.40 (10.40, 12.30) |
| Hemoglobin DCM g/dl, Median (IQR) | 10.75 (10.20, 11.50) | 10.80 (10.00, 11.50) | 10.40 (9.60, 11.10) | 10.60 (10.00, 11.40) |
| Anemia (HemoCue), n (%) (Yes) | 34 (25.0%) | 7 (21.2%) | 34 (33.3%) | 75 (27.7%) |
| Anemia (DCM), n (%) (Yes) | 45 (33.1%) | 12 (36.4%) | 53 (52.0%) | 110 (40.6%) |

Abbreviations: IQR- Inter quartile range, SD- Standard Deviation, DCM- direct cyanmethemoglobin method
