# Supplementary material for: Validation of HemoCue method and development of a revised hemoglobin cut-off to detect anemia in children aged 6–24-months
Source: PLoS One. 2026 Oct 16;21(10):e0346506. doi: 10.1371/journal.pone.0346506 (PMC13674700; doi:10.1371/journal.pone.0346506)
Supplement: Supplementary file 15 [file pone.0346506.s015.docx]

**S14 Table. Different hemoglobin cut-off metric for stunted (length-for-age ≤ –2 SD) children**

| Metric | Cut-point 10.80 | Cut-point 10.90 | Cut-point 11.00 | Cut-point 11.10 |
| --- | --- | --- | --- | --- |
| Accuracy | 80.44% | 78.23% | 78.97% | 77.12% |
| Kappa | 58.43% | 54.27% | 56.33% | 52.97% |
| Mcnemar’s Test (p-value) | 0.028 | 0.298 | >0.999 | 0.525 |
| Precision | 0.807 | 0.753 | 0.743 | 0.707 |
| Sensitivity (True Positive Rate) | 68.18% | 69.09% | 73.64% | 74.55% |
| Specificity (True Negative Rate) | 88.82% | 84.47% | 82.61% | 78.88% |
| Positives predicted value | 80.65% | 75.25% | 74.31% | 70.69% |
| Negatives predicted value | 80.34% | 80.00% | 82.10% | 81.94% |
| Detection Rate | 27.68% | 28.04% | 29.89% | 30.26% |
| Balanced Accuracy | 78.50% | 76.78% | 78.12% | 76.71% |

Abbreviations: SD- Standard Deviation
