# Supplementary material for: Validation of HemoCue method and development of a revised hemoglobin cut-off to detect anemia in children aged 6–24-months
Source: PLoS One. 2026 Oct 16;21(10):e0346506. doi: 10.1371/journal.pone.0346506 (PMC13674700; doi:10.1371/journal.pone.0346506)
Supplement: Supplementary file 17 [file pone.0346506.s017.docx]

**S16 Table. Underweight (weight-for-age ≤ –2 SD) participants baseline characteristics**

| Indicator | Case | Control | Cohort | Overall |
| --- | --- | --- | --- | --- |
| Number of samples | 148 | 3 | 66 | 217 |
| Age (month), Median (IQR) | 14.00 (11.00, 18.00) | 17.00 (12.00, 19.00) | 15.00 (7.00, 15.00) | 15.00 (10.00, 16.00) |
| Sex, n (%) (Girl) | 77 (52.0%) | 1 (33.3%) | 35 (53.0%) | 113 (52.1%) |
| Length-for-age z score, Mean (SD) | -2.72 (0.89) | -1.95 (0.57) | -2.55 (0.90) |  |
| Weight-for-age z score, Mean (SD) | -2.77 (0.60) | -2.15 (0.06) | -2.61 (0.55) |  |
| Weight-for-length z score, Mean (SD) | -1.79 (0.71) | -1.59 (0.38) | -1.67 (0.71) |  |
| Hemoglobin HemoCue g/dl, Median (IQR) | 11.60 (10.60, 12.45) | 11.20 (8.90, 14.40) | 10.85 (10.10, 11.90) | 11.40 (10.40, 12.30) |
| Hemoglobin DCM g/dl, Median (IQR) | 10.80 (10.25, 11.50) | 10.50 (8.40, 12.80) | 10.15 (9.60, 11.10) | 10.60 (10.00, 11.40) |
| Anemia (HemoCue), n (%) (Yes) | 32 (21.6%) | 1 (33.3%) | 23 (34.8%) | 56 (25.8%) |
| Anemia (DCM), n (%) (Yes) | 49 (33.1%) | 1 (33.3%) | 37 (56.1%) | 87 (40.1%) |

Abbreviations: IQR- Inter quartile range, SD- Standard Deviation, DCM- direct cyanmethemoglobin method
