# Supplementary material for: Validation of HemoCue method and development of a revised hemoglobin cut-off to detect anemia in children aged 6–24-months
Source: PLoS One. 2026 Oct 16;21(10):e0346506. doi: 10.1371/journal.pone.0346506 (PMC13674700; doi:10.1371/journal.pone.0346506)
Supplement: Supplementary file 18 [file pone.0346506.s018.docx]

**S17 Table. Different hemoglobin cut-off metric for underweight (weight-for-age ≤ –2 SD) children**

| Metric | Cut-point 10.80 | Cut-point 10.90 | Cut-point 11.00 | Cut-point 11.10 |
| --- | --- | --- | --- | --- |
| Accuracy | 81.57% | 79.72% | 80.18% | 78.80% |
| Kappa | 60.73% | 57.30% | 58.83% | 56.37% |
| Mcnemar’s Test (p-value) | 0.082 | 0.451 | >0.999 | 0.461 |
| Precision | 0.813 | 0.765 | 0.750 | 0.720 |
| Sensitivity (True Positive Rate) | 70.11% | 71.26% | 75.86% | 77.01% |
| Specificity (True Negative Rate) | 89.23% | 85.38% | 83.08% | 80.00% |
| Positives predicted value | 81.33% | 76.54% | 75.00% | 72.04% |
| Negatives predicted value | 81.69% | 81.62% | 83.72% | 83.87% |
| Detection Rate | 28.11% | 28.57% | 30.41% | 30.88% |
| Balanced Accuracy | 79.67% | 78.32% | 79.47% | 78.51% |

Abbreviations: SD- Standard Deviation
