# Supplementary material for: Validation of HemoCue method and development of a revised hemoglobin cut-off to detect anemia in children aged 6–24-months
Source: PLoS One. 2026 Oct 16;21(10):e0346506. doi: 10.1371/journal.pone.0346506 (PMC13674700; doi:10.1371/journal.pone.0346506)
Supplement: Supplementary file 20 [file pone.0346506.s020.docx]

**S19 Table. Participants baseline characteristics based on acute systemic inflammation status using C-reactive protein (CRP) cut-off of ≥ 5 mg/L**

| Indicator | Case | Control | Cohort | Overall |
| --- | --- | --- | --- | --- |
| Number of samples | 38 | 35 | 107 | 180 |
| Age (month), Median (IQR) | 14.00 (11.00, 19.00) | 12.00 (10.00, 19.00) | 15.00 (7.00, 15.00) | 14.00 (7.00, 15.00) |
| Sex, n (%) (Girl) | 25 (65.8%) | 16 (45.7%) | 55 (51.4%) | 96 (53.3%) |
| Length-for-age z score, Mean (SD) | -2.85 (1.00) | -1.11 (0.93) | -1.66 (1.07) |  |
| Weight-for-age z score, Mean (SD) | -2.91 (0.75) | -0.50 (0.76) | -1.25 (1.10) |  |
| Weight-for-length z score, Mean (SD) | -1.89 (0.88) | 0.11 (0.94) | -0.49 (1.06) |  |
| Hemoglobin HemoCue g/dl, Median (IQR) | 11.80 (10.70, 12.60) | 11.90 (10.00, 12.80) | 10.90 (10.30, 11.90) | 11.20 (10.30, 12.35) |
| Hemoglobin DCM g/dl, Median (IQR) | 10.85 (10.50, 11.50) | 10.60 (9.20, 11.40) | 10.40 (9.60, 11.20) | 10.50 (9.60, 11.30) |
| Anemia (HemoCue), n (%) (Yes) | 6 (15.8%) | 11 (31.4%) | 36 (33.6%) | 53 (29.4%) |
| Anemia (DCM), n (%) (Yes) | 9 (23.7%) | 15 (42.9%) | 55 (51.4%) | 79 (43.9%) |

Abbreviations: IQR- Inter quartile range, SD- Standard Deviation, DCM- direct cyanmethemoglobin method
