# Supplementary material for: Validation of HemoCue method and development of a revised hemoglobin cut-off to detect anemia in children aged 6–24-months
Source: PLoS One. 2026 Oct 16;21(10):e0346506. doi: 10.1371/journal.pone.0346506 (PMC13674700; doi:10.1371/journal.pone.0346506)
Supplement: Supplementary file 21 [file pone.0346506.s021.docx]

**S20 Table. Different hemoglobin cut-off metric for acute systemic inflammation status using C-reactive protein (CRP) cut-off of ≥ 5 mg/L**

| Metric | Cut-point 10.80 | Cut-point 10.90 | Cut-point 11.00 | Cut-point 11.10 |
| --- | --- | --- | --- | --- |
| Accuracy | 81.11% | 81.11% | 80.00% | 79.44% |
| Kappa | 61.11% | 61.33% | 59.51% | 58.44% |
| Mcnemar’s Test (p-value) | 0.123 | 0.391 | 0.868 | 0.742 |
| Precision | 0.826 | 0.808 | 0.765 | 0.756 |
| Sensitivity (True Positive Rate) | 72.15% | 74.68% | 78.48% | 78.48% |
| Specificity (True Negative Rate) | 88.12% | 86.14% | 81.19% | 80.20% |
| Positives predicted value | 82.61% | 80.82% | 76.54% | 75.61% |
| Negatives predicted value | 80.18% | 81.31% | 82.83% | 82.65% |
| Detection Rate | 31.67% | 32.78% | 34.44% | 34.44% |
| Balanced Accuracy | 80.14% | 80.41% | 79.83% | 79.34% |
