# Supplementary material for: Validation of HemoCue method and development of a revised hemoglobin cut-off to detect anemia in children aged 6–24-months
Source: PLoS One. 2026 Oct 16;21(10):e0346506. doi: 10.1371/journal.pone.0346506 (PMC13674700; doi:10.1371/journal.pone.0346506)
Supplement: Supplementary file 22 [file pone.0346506.s022.docx]

**S21 Table. Anemia detection in test dataset with new and old cut-offs for acute systemic inflammation status using C-reactive protein (CRP) cut-off of ≥ 5 mg/L**

| Anemia detection methods and cut-off (n= 55) | **%** |
| --- | --- |
| Anemic using DCM (cut-off 10.5 g/dL) | 49.1 |
| Anemic using HemoCue (cut-off 10.5 g/dL) | 29.1 |
| Anemic using HemoCue (cut-off 11.00 g/dL) | 40.0 |

Abbreviations: DCM- direct cyanmethemoglobin method
