# Supplementary material for: Validation of HemoCue method and development of a revised hemoglobin cut-off to detect anemia in children aged 6–24-months
Source: PLoS One. 2026 Oct 16;21(10):e0346506. doi: 10.1371/journal.pone.0346506 (PMC13674700; doi:10.1371/journal.pone.0346506)
Supplement: Supplementary file 23 [file pone.0346506.s023.docx]

**S22 Table. Participants baseline characteristics based on acute systemic inflammation status using Alpha-1-Acid Glycoprotein (AGP) cut-off of ≥ 100 mg/dL**

| Indicator | Case | Control | Cohort | Overall |
| --- | --- | --- | --- | --- |
| Number of samples | 98 | 133 | 120 | 351 |
| Age (month), Median (IQR) | 14.00 (11.00, 17.00) | 13.00 (9.00, 17.00) | 15.00 (7.00, 15.00) | 13.00 (8.00, 15.00) |
| Sex, n (%) (Girl) | 49 (50.0%) | 53 (39.8%) | 63 (52.5%) | 165 (47.0%) |
| Length-for-age z score, Mean (SD) | -2.71 (0.87) | -1.08 (0.85) | -1.68 (1.03) |  |
| Weight-for-age z score, Mean (SD) | -2.73 (0.73) | -0.58 (0.70) | -1.34 (1.10) |  |
| Weight-for-length z score, Mean (SD) | -1.76 (0.85) | -0.02 (0.76) | -0.58 (1.06) |  |
| Hemoglobin HemoCue g/dl, Median (IQR) | 11.70 (10.60, 12.40) | 11.90 (10.90, 12.70) | 10.90 (10.20, 12.00) | 11.50 (10.40, 12.40) |
| Hemoglobin DCM g/dl, Median (IQR) | 10.80 (10.30, 11.50) | 10.90 (9.80, 11.40) | 10.40 (9.60, 11.10) | 10.70 (10.00, 11.40) |
| Anemia (HemoCue), n (%) (Yes) | 21 (21.4%) | 27 (20.3%) | 40 (33.3%) | 88 (25.1%) |
| Anemia (DCM), n (%) (Yes) | 29 (29.6%) | 48 (36.1%) | 61 (50.8%) | 138 (39.3%) |

Abbreviations: IQR- Inter quartile range, SD- Standard Deviation, DCM- direct cyanmethemoglobin method
