# Supplementary material for: Validation of HemoCue method and development of a revised hemoglobin cut-off to detect anemia in children aged 6–24-months
Source: PLoS One. 2026 Oct 16;21(10):e0346506. doi: 10.1371/journal.pone.0346506 (PMC13674700; doi:10.1371/journal.pone.0346506)
Supplement: Supplementary file 24 [file pone.0346506.s024.docx]

**S23 Table. Different hemoglobin cut-off metric for acute systemic inflammation status using Alpha-1-Acid Glycoprotein (AGP) cut-off of ≥ 100 mg/dL**

| Metric | Cut-point 10.80 | Cut-point 10.90 | Cut-point 11.00 | Cut-point 11.10 |
| --- | --- | --- | --- | --- |
| Accuracy | 81.20% | 80.06% | 80.91% | 79.20% |
| Kappa | 59.13% | 57.22% | 59.64% | 56.36% |
| Mcnemar’s Test (p-value) | <0.000 | 0.042 | 0.464 | >0.999 |
| Precision | 0.827 | 0.783 | 0.771 | 0.737 |
| Sensitivity (True Positive Rate) | 65.94% | 68.12% | 73.19% | 73.19% |
| Specificity (True Negative Rate) | 91.08% | 87.79% | 85.92% | 83.10% |
| Positives predicted value | 82.73% | 78.33% | 77.10% | 73.72% |
| Negatives predicted value | 80.50% | 80.95% | 83.18% | 82.71% |
| Detection Rate | 25.93% | 26.78% | 28.77% | 28.77% |
| Balanced Accuracy | 78.51% | 77.95% | 79.55% | 78.14% |
