# Supplementary material for: Validation of HemoCue method and development of a revised hemoglobin cut-off to detect anemia in children aged 6–24-months
Source: PLoS One. 2026 Oct 16;21(10):e0346506. doi: 10.1371/journal.pone.0346506 (PMC13674700; doi:10.1371/journal.pone.0346506)
Supplement: Supplementary file 25 [file pone.0346506.s025.docx]

**S24 Table. Anemia detection in test dataset with new and old cut-offs for acute systemic inflammation status using Alpha-1-Acid Glycoprotein (AGP) cut-off of ≥ 100 mg/dL**

| Anemia detection methods and cut-off (n= 107) | **%** |
| --- | --- |
| Anemic using DCM (cut-off 10.5 g/dL) | 37.4 |
| Anemic using HemoCue (cut-off 10.5 g/dL) | 26.2 |
| Anemic using HemoCue (cut-off 11.00 g/dL) | 40.2 |

Abbreviations: DCM- direct cyanmethemoglobin method
